# CobraMod: a pathway-centric curation tool for constraint-based metabolic models

**DOI:** 10.1093/bioinformatics/btac119

**Published:** 2022-02-24

**Authors:** Stefano Camborda, Jan-Niklas Weder, Nadine Töpfer

**Affiliations:** Independent Research Group, Molecular Genetics Department, Leibniz Institute of Plant Genetics and Crop Plant Research, Gatersleben, Germany; Independent Research Group, Molecular Genetics Department, Leibniz Institute of Plant Genetics and Crop Plant Research, Gatersleben, Germany; Independent Research Group, Molecular Genetics Department, Leibniz Institute of Plant Genetics and Crop Plant Research, Gatersleben, Germany

## Abstract

**Summary:**

COnstraint-Based Reconstruction and Analysis of genome-scale metabolic models has become a widely used tool to understand metabolic network behavior at a large scale. However, existing reconstruction tools lack functionalities to address modellers' common objective to study metabolic networks on the pathway level. Thus, we developed CobraMod—a Python package for pathway-centric modification and extension of genome-scale metabolic networks. CobraMod can integrate data from various metabolic pathway databases as well as user-curated information. Our tool tests newly added metabolites, reactions and pathways against multiple curation criteria, suggests manual curation steps and provides the user with records of changes to ensure high quality metabolic reconstructions. CobraMod uses the visualization tool Escher for pathway representation and offers simple customization options for comparison of pathways and flux distributions. Our package enables coherent and reproducible workflows as it can be seamlessly integrated with COBRApy and Escher.

**Availability and implementation:**

The source code can be found at https://github.com/Toepfer-Lab/cobramod/ and can be installed with pip. The documentation including tutorials is available at https://cobramod.readthedocs.io/.

## 1 Introduction

Genome-scale metabolic models (GEMs) and their analysis by constraint-based modeling techniques are widely used tools to study metabolic systems at a large scale. Several software tools for Constraint-Based Reconstruction and Analysis (COBRA) are available, such as the COBRA toolbox, ModelSEED, Pathway Tools, RAVEN, CarveMe and Merlin (Dias *et al.*, 2015; Heirendt *et al.*, 2019; Karp, 2002; Machado *et al.*, 2018; Seaver *et al.*, 2021; Wang *et al.*, 2018) and have been evaluated here (Faria *et al.*, 2018; Mendoza *et al.*, 2019). In recent years, the freely available and community-supported software package COBRApy has gained particular popularity (Ebrahim *et al.*, 2013). COBRApy performs commonly used COBRA methods such as flux balance analysis, flux variability analysis, gene deletion analysis and includes simple, object-oriented interfaces for model reconstruction.

Several software packages complement COBRApy by implementing extended functionalities. For instance, Cameo and MewPy offer functionalities for computational strain optimization, MEMOTE includes a suite of tests to assess GEM quality, Medusa facilitates generating and analyzing ensembles of GEMs, and the Escher visualization tool offers an user-friendly interface for designing and manipulating pathway maps (Lieven, 2020; Cardoso *et al.*, 2018; King *et al.*, 2015; Medlock *et al.*, 2020; Pereira *et al.*, 2021). However, currently available reconstruction tools rely either on error-prone, automated reconstruction procedures or laborious, manual addition of individual reactions or reaction sets and thus preclude the extension and curation of GEMs based on their biologically meaningful subsets, i.e. the metabolic pathways.

Here, we present CobraMod, a pathway-centric curation tool for the modification and extension of GEMs. CobraMod offers a comprehensible set of functions for semi-automated network extension, testing and visualization and enables easy, user-friendly manual curation and information logging to ensure high quality network reconstructions. CobraMod is written in Python 3; it builds upon and extends COBRApy and can directly interact with Escher for pathway and flux visualization.

## 2 Implementation

**Fig. 1.**
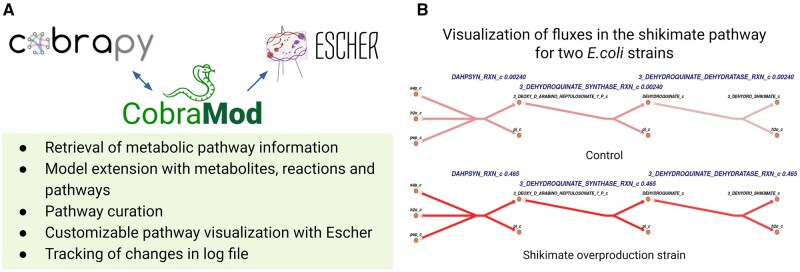
CobraMod’s main functionalities and pathway visualization example. (**A**) CobraMod’s pathway-centric functionalities bridge COBRApy methods and the visualization tool Escher. (**B**) Visualization of a metabolic engineering case study of the shikimate pathway in *E.coli*. Flux solutions for two strains of *E.coli* (control and engineered) are visualized. For simplicity, we represented only three reactions of the whole pathway. Reaction names and pathway fluxes are given in blue. For comparability, flux values were normalized and darker colors indicate higher flux values

CobraMod is an open-source package which enables modifying and extending GEMs with metabolic pathway information from various databases or user-curated datasets. Our package converts these data into native COBRApy objects and quality-checks them for multiple curation criteria before incorporating them into the model (Fig. 1A). CobraMod’s main functions include downloading metabolic pathway information (get_data), creating COBRApy objects (create_objects) and including new metabolites (add_metabolites), reactions (add_reactions) or pathways (add_pathway), as well as testing a reaction’s capability to carry a non-zero flux (test_non_zero_flux) and pathway visualization (visualize).

### 2.1 Data retrieval

CobraMod supports all databases from the BioCyc collection (Karp *et al.*, 2019), the KEGG database (Kanehisa and Goto, 2000) and the BiGG Models repository (King *et al.*, 2016). The user can retrieve metabolic pathway information by specifying a database and the corresponding identifiers for metabolites, reactions or pathways. CobraMod automatically gathers gene information when obtaining information for reactions or pathways. CobraMod then downloads these datasets, stores them locally to ensure reproducibility (get_data), and transforms them into COBRApy objects (create_object). In addition, CobraMod can integrate user-curated metabolites and reactions via text file or direct script input (add_metabolites, add_reactions).

### 2.2 Curation steps

CobraMod enables modifying and analyzing GEMs on the metabolic pathway level. Thus, it combines sets of reactions into pathway-objects, which the user can directly add to the model (add_pathways). Reactions and metabolites of a given pathway-object will undergo a curation process in which they are tested for duplicate elements, missing chemical formulas of the metabolites, mass balance of reactions and reaction reversibility (detailed in the documentation). To ensure that the added pathways are functional we implemented a non-zero flux test (test_non_zero_flux). During the test, CobraMod can add auxiliary source reactions and suggests manual curation steps based on these auxiliary modifications. Moreover, CobraMod offers cross-referencing and meta-data curation and is MEMOTE-compliant. Our tool offers comprehensible and user-friendly tracking of the curation process. When a pathway-object is added to the model a summary is outputted and the complete curation procedure is written to a log file. If any of the curation criteria is not met or exceptions are encountered, CobraMod passes a warning through the Python console and the log file.

### 2.3 Visualization

CobraMod uses Escher for pathway visualization. To this end, each pathway-object includes a visualization method (visualize) which automatically generates pathway maps of the respective set of reactions. These pathway maps can be easily customized to visualize flux distributions using default or user-defined colors and gradients (linear or quantile normalized).

## 3 Test case

To demonstrate CobraMod’s functionalities we implemented two test cases based on *in vivo* and *in silico* overproduction studies in *Escherichia coli*. In the first example, we used a core model of *E.coli* (Orth *et al.*, 2010) to reproduce engineering strategies for improved shikimate synthesis (Chen *et al.*, 2014). Using our Escher interface, we visualized shikimate production for the control and one of the engineered strains (Fig. 1B). In a second example, we utilize a genome-scale model of *E.coli* (Monk *et al.*, 2017) to reproduce *in silico* experiments that introduce a synthetic homoserine cycle as an efficient route for methylotrophic growth (He *et al.*, 2020) and demonstrate the strength of CobraMod’s pathway-centric curation procedures. The test cases with a step-by-step workflow can be found in the documentation.

## 4 Conclusion

CobraMod offers user-friendly, pathway-centric extension, curation and flux visualization for large-scale metabolic networks. It thus addresses a common modeller's objective to study metabolic network behavior on the pathway level. CobraMod employs as much automation as possible and suggests necessary manual curation steps to ensure high quality metabolic reconstructions. Our tool can be directly linked with COBRApy and the Escher visualization tool and thus enables coherent and reproducible workflows.
